# Supplementary material for: Differences in COVID-19 Risk by Race and County-Level Social Determinants of Health among Veterans
Source: Int J Environ Res Public Health. 2021 Dec 13;18(24):13140. doi: 10.3390/ijerph182413140 (PMC8701661; doi:10.3390/ijerph182413140)
Supplement: Supplementary file 1 [file ijerph-18-13140-s001.zip › ijerph-1474332-supplementary.pdf]

Supplementary Table S1. Data Dictionary for county-level socioeconomic measures.

Measure	Source	Source Details	Original Variable
Percentage of Persons in Deep Poverty, 2017	Area Health Resources Files	(.1) Table B17024 American Community Survey 2013-2017	F15419-13
Percentage Persons 65+ in Deep Poverty, 2017	Area Health Resources Files	(.1) Table B17024 American Community Survey 2013-2017	F15425-13
Percentage without High School Diploma, Ages 25+, 2013-2017 (5-year)	Centers for Disease Control and Prevention	American Community Survey 2013-2017	prop_edu_less_highschool
Percentage without 4+ Years College, Ages 25+, 2013-2017 (5-year)	Centers for Disease Control and Prevention	American Community Survey 2013-2017	prop_edu_less_college
Percentage Food Stamp/Supplemental Nutrition Assistance Program Recipients, 2015	Centers for Disease Control and Prevention	Health Resources and Services Administration; Area Health Resources File	prop_food_stamp_sp_recip
Percentage Living in Poverty, All Ages, 2016	Centers for Disease Control and Prevention	U.S. Census Bureau (SAIPE); Small Area Income and Policy Estimates	prop_poverty
Percentage without Health Insurance, Under Age 65, 2016	Centers for Disease Control and Prevention	U.S. Census Bureau (SAHIE) Small Area Health Insurance Estimates	prop_uninsured
Percentage Eligible for Medicaid, All Ages, 2012	Centers for Disease Control and Prevention	Health Resources and Services Administration; Area Health Resources File	prop_medicaid_eligible
Percentage in Crowded Housing, 2013-2017	Diversity Data for Kids	Diversity Data Kids (25014_1_P_050_5_crowded_housing_race)	total_est
Percentage 65+ living alone, 2018	US Census Bureau	American Community Survey 5-year 2018 Table DP02	DP02_0012PE
Percentage of households where grandparent have children under 18, 2018	US Census Bureau	American Community Survey 5-year 2018 Table DP02	DP02_0043PE
Percentage of Households without a computer, 2018	US Census Bureau	American Community Survey 5-year 2018 Table DP02	DP02_0151PE
Percentage of households without broadband, 2018	US Census Bureau	American Community Survey 5-year 2018 Table DP02	DP02_0152PE
Percentage of US Non-Native Residents, 2018	US Census Bureau	American Community Survey 5-year 2018 Table DP02	DP02_0087PE
Percentage Non-White, 2018	US Census Bureau	American Community Survey 5-year 2018 Table DP02	DP02_0087E
Median Household Income (thousands), 2016	Centers for Disease Control and Prevention	U.S. Census Bureau (SAIPE); Small Area Income and Policy Estimates	NA
Income Inequality (GINI Index), 2018	US Census Bureau	American Community Survey 5-year 2018	B19083_001E
Unemployment Rate Ages 16+, 2017	Centers for Disease Control and Prevention	U.S. Bureau of Labor Statistics; Local Area Unemployment Statistics	unemployment_rate

[1] Crowded housing defined as the number of occupied housing units with more than one occupant per room divided by the number of occupied housing units, times 100, for the total population and by race/ethnicity. [2] Data were retrieved from the American Community Survey, U.S. Census Bureau, U.S. Bureau of Labor Statistics, and Diversity Data for Kids as previously described in Chin et al 2020, and available at https://bmjopen.bmj.com/content/10/9/e039886.

Supplementary Table S2. Correlation between County Level Social Determinants of Health.

Variables	1	2	3	4	5	6	7	8	9	10	11	12	13	14	15	16	17	18
1) Persons in Deep Poverty	1.00																	
2) Persons 65+ in Deep Poverty	0.44	1.00																
3) Without High School Diploma	0.53	0.38	1.00															
4) Without 4+ Years College	0.35	0.24	0.71	1.00														
5)Food Stamp/SNAP Recipients	0.71	0.40	0.66	0.59	1.00													
6) Living in Poverty, All Ages	0.84	0.40	0.73	0.57	0.83	1.00												
7) Without Health Insurance	0.33	0.26	0.60	0.42	0.33	0.44	1.00											
8) Eligible for Medicaid, All Age	0.60	0.36	0.63	0.56	0.84	0.73	0.20	1.00										
9) Living in Crowded Housing	0.20	0.19	0.41	0.11	0.19	0.22	0.37	0.34	1.00									
10) 65+ living alone	0.22	0.16	0.24	0.39	0.36	0.35	0.10	0.32	-0.20	1.00								
11) Households with children <1	0.28	0.27	0.60	0.43	0.41	0.39	0.46	0.40	0.48	-0.08	1.00							
12) Households w/out Computer	0.58	0.39	0.71	0.71	0.69	0.75	0.37	0.61	0.06	0.55	0.33	1.00						
13) Households w/out broadband	0.61	0.39	0.73	0.73	0.69	0.77	0.46	0.61	0.09	0.49	0.38	0.94	1.00					
14) Non-USA Native	-0.16	0.03	-0.03	-0.48	-0.23	-0.22	0.05	-0.09	0.52	-0.32	0.11	-0.40	-0.41	1.00				
15) Non-White	0.44	0.33	0.47	0.06	0.47	0.48	0.43	0.41	0.53	-0.10	0.50	0.24	0.28	0.44	1.00			
16) Median Household Income	-0.67	-0.30	-0.65	-0.76	-0.75	-0.83	-0.45	-0.67	-0.06	-0.50	-0.26	-0.74	-0.77	0.45	-0.17	1.00		
17) Income Inequality (Gini)	0.60	0.33	0.32	-0.01	0.46	0.57	0.25	0.43	0.16	0.28	0.11	0.36	0.36	0.10	0.40	-0.40	1.00	
18) Unemployment Rate	0.46	0.30	0.50	0.44	0.58	0.57	0.28	0.54	0.23	0.27	0.39	0.49	0.46	-0.08	0.41	-0.46	0.29	1.00
